# Tip-Clearance Measurement in the First Stage of the Compressor of an Aircraft Engine

**DOI:** 10.3390/s16111897

**Published:** 2016-11-11

**Authors:** Iker García, Radosław Przysowa, Josu Amorebieta, Joseba Zubia

**Affiliations:** 1 Department of Communications Engineering, Engineering School of Bilbao, University of the Basque Country UPV/EHU, Alda. Urquijo s/n E-48013 Bilbao, Spain; jamorebieta001@ikasle.ehu.eus (J.A.); joseba.zubia@ehu.eus (J.Z.); 2 Air Force Institute of Technology (Instytut Techniczny Wojsk Lotniczych), ul. Ksiecia Boleslawa 6 01-494 Warszawa, Poland; radoslaw.przysowa@itwl.pl

**Keywords:** tip clearance, optical fiber sensor, aircraft engine, optical fiber bundle, compressor

## Abstract

In this article, we report the design of a reflective intensity-modulated optical fiber sensor for blade tip-clearance measurement, and the experimental results for the first stage of a compressor of an aircraft engine operating in real conditions. The tests were performed in a ground test cell, where the engine completed four cycles from idling state to takeoff and back to idling state. During these tests, the rotational speed of the compressor ranged between 7000 and 15,600 rpm. The main component of the sensor is a tetrafurcated bundle of optical fibers, with which the resulting precision of the experimental measurements was 12 µm for a measurement range from 2 to 4 mm. To get this precision the effect of temperature on the optoelectronic components of the sensor was compensated by calibrating the sensor in a climate chamber. A custom-designed MATLAB program was employed to simulate the behavior of the sensor prior to its manufacture.

## 1. Introduction

The development of more and more efficient aircraft engines is a continuous challenge that provides several benefits. In addition to monetary profit, carbon-emission reductions, longer service lives, and flight-range capabilities of the aeroplanes are improved, thanks to the reduction of fuel burnt [1]. The performance of the engine can be significantly improved by minimizing the leak flows through the gap between the blade tip and the casing of the compressor or the turbine. Therefore, this distance, known as tip clearance (TC), plays a major role in the aerodynamic efficiency of axial compressors and turbines [2]. The TC value varies with the operation condition of the engine (ground idle, takeoff, cruise, and landing) [3], as well as with the engine aging [4]. These fluctuations of the TC are due to two types of loads, namely engine and flight loads. The first kind of load encompasses centrifugal, thermal, internal engine pressure, and thrust loads, whereas flight loads are comprised by inertial (gravitational), aerodynamic (external pressure), and gyroscopic loads [5].

An accurate and real-time TC measurement is necessary to prevent any blade contacting with the casing and to lessen the leak flows in the fan, compressor, and turbine sections of the engines, which serves to optimize the engine performance. That is, precisely, the purpose of active-clearance control systems, in which the TC is limited by directing air to the casing by means of valves to control the thermal expansion of the casing, and to keep the TC to a minimum so that the engine efficiency increases. In contrast with power-system turbines where TC common values range from 2 to 8 mm, in aircraft turbines TC values are usually lower than 3 mm [6], so a resolution better than 25 µm is required for the whole measurement interval [7]. Currently, several kinds of sensors are employed to carry out TC measurements. The most common method is the employment of capacitive sensors [8,9]. These sensors are robust, small, and low-cost, but their accuracy is limited to 30 µm [10]. Microwave sensors have also been employed for TC measurements [11,12]. Since their signal depends on several variables, they require a complex calibration and advanced processing of signals. Besides, they provide a limited spatial resolution as compared to optical sensors, and it is not an economical technology [13]. Another option are eddy-current (inductive) sensors, which have the advantage of not requiring a direct view of the blade tip, so the sensor is not exposed to the harsh conditions of the engine [14], but their calibration is highly dependent on the tip shape and temperature [15]. Finally, optical sensors offer multiple inherent advantages [16] and that is the reason why they are being more and more employed in the aircraft industry [17,18,19]. Regarding TC measurement, optical sensors yield the best resolution. On the contrary, they are seriously affected by contamination and debris, thus being suitable only for the clean parts of the engine such as the fan and the compressor, or for the testing of rigs during the engine-development phase [20].

Optical sensors for TC measurements have been developed by using diverse techniques such as Doppler positioning [21] or interferometry [22]. Nevertheless, the simplest and most affordable devices to obtain high accuracy [23] and bandwidth are intensity-based sensors [24,25]. Several configurations of intensity-modulated sensors using trifurcated bundles for TC measurements have been previously proposed by other authors [15,26,27]. However, their performances were demonstrated only in laboratory conditions and using a rig instead of a real engine. In addition to this, their measurement ranges and stand-off distances are not suitable for measurements in real engines. In this paper, we present the results obtained for an intensity-based sensor whose principal component is a tetrafurcated bundle of optical fibers. It was specifically designed to carry out tip clearance and tip timing measurements in a compressor of a real engine in a simultaneous and independent way. In Section 2, the experimental set-up and the sensor design are explained. In Section 3, the results obtained in the compressor of a real aircraft engine are presented and discussed. The conclusions of the work are summarized in Section 4.

## 2. Materials and Methods

### 2.1. Sensor Design

**Figure 1:**
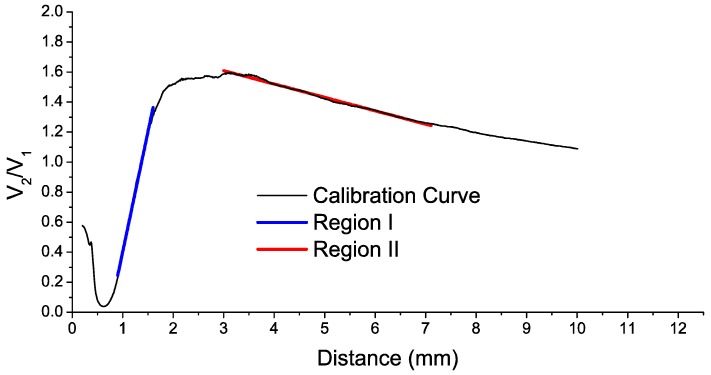
Typical calibration curve for a sensor using a trifurcated bundle of optical fibers.

In previous works, we developed a reflective intensity-modulated optical fiber sensor to carry out TC measurements in turbines [20] and rotating components of aircraft engines [28]. The essential component of this sensor was a bundle of optical fibers. We used to employ trifurcated bundles, with one common leg on one side and three independent legs on the other. One of these legs was connected to a light source (illuminating fiber), and the other two legs were connected to their respective photodetectors (receiving fibers). The illuminating fiber was located in the center of the common leg and the light emitted by this fiber was reflected by the target and collected by two rings of receiving fibers surrounding the illuminating fiber. Each ring of receiving fibers was gathered into a leg on the other end of the bundle, which was connected to a photodetector in order to convert the optical signals into an electrical one. Finally, the quotient of these two voltage signals (V_1_ and V_2_) was related to the distance of the target by a linearized calibration curve as the one shown in Figure 1 (for a more detailed explanation of the sensor operation see [20,29]).

With respect to the works of other groups, we introduced two important improvements in the sensor design [29]. Firstly, we used a single-mode fiber as illuminating fiber to reduce the modal noise at the output of the bundle. The second improvement was the employment of asymmetric gain for the photodetectors, which increases the sensitivity of the sensor. The resulting sensitivity was more than the double of the sensitivity obtained using a configuration of symmetric gain as it is shown in [29]. In the calibration curve depicted in Figure 1, two different regions can be distinguished. The region I (front-slope region) provides more sensitivity than the region II (back-slope region). In addition, the region I is less sensitive to noise since the amplitudes of V1 and V2 have higher values than those of the region II. On the other hand, the measurement range from 1 to 1.6 mm may be too short and it requires placing the bundle tip very close to the blades. In previous tests, we employed the region II due to these constraints. However, in this occasion we decided to make some changes in the bundle design so that the most sensitive region I could be used for the tests.

**Figure 2:**
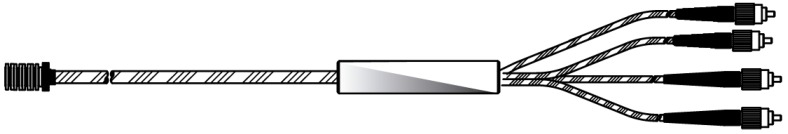
Tetrafurcated bundle employed in the TC optical sensor.

Once the sensor was assembled in the casing of the engine, the required measurement range for the TC was estimated to be the interval from 2 to 4 mm. Therefore, the first necessary variation in the bundle design consists in shifting the measurement range so that it starts at 2 mm. The beginning of region I is determined by the target distance in which the reflected light starts to enter the second ring of receiving fibers, so the distance between the center of the illuminating fiber and the fibers of the second ring of receiving fibers was increased in the new design. To take this ring away, we could have chosen the option of inserting a considerable number of needles between the first and second rings of receiving fibers until achieving the required distance, but we decided to insert fewer needles and to introduce another ring of receiving fibers. The fibers of this ring were gathered in another independent leg, so what we have is a tetrafurcated bundle (see Figure 2). This leg allows carrying out tip-timing measurements with another photodetector whose gain, and therefore whose bandwidth, is not dependent on the gain of the photodetectors used for the TC configuration [30]. The common leg of the bundle has a threaded head to facilitate its coupling to the casing of the engine, whereas the legs on the other side have conventional FC connectors.

**Figure 3:**
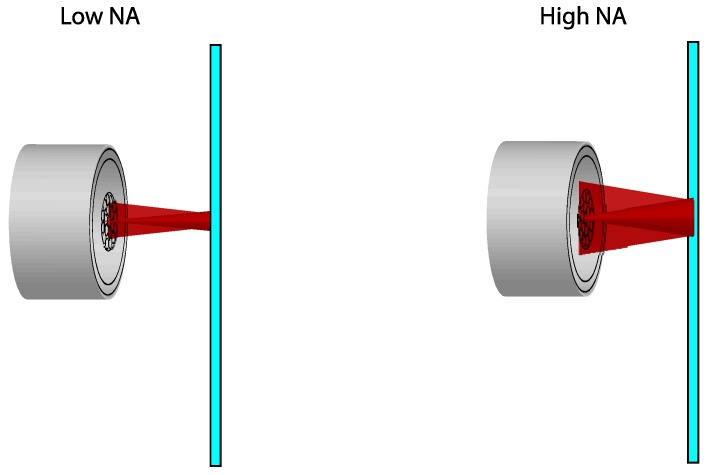
Effect of the variation of the numerical aperture of the illuminating fibers over the reflected-light cone.

**Figure 4:**
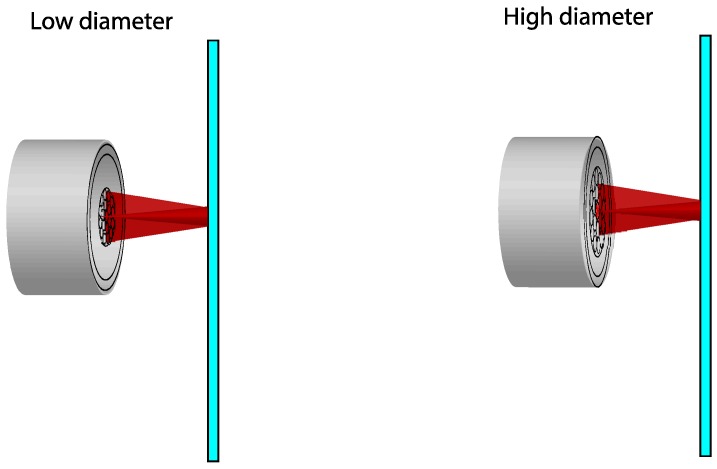
Effect of the variation of the diameter of the receiving fibers.

The second necessary variation in the bundle design consists in widening the measurement range of the region I. We could modify two characteristics of the optical fibers in order to get this objective. The first option is to use an illuminating fiber with a lower numerical aperture. The upper limit of the measurement range for the region I is determined by the distance of the target in which the reflected-light cone completely covers the second ring of receiving fibers. Thus, the measurement range becomes higher as the numerical aperture of the illuminating fiber becomes smaller, as illustrated in Figure 3. Since a single-mode fiber with a numerical aperture of 0.12 is employed as illuminating fiber, there is little margin to reduce it. The other option to extend the range is to increment the diameter of the receiving fibers so that the target distance can be greater before the second ring is completely covered by the reflected light. The effect of increasing the diameter of the receiving fibers is depicted in Figure 4.

**Figure 5:**
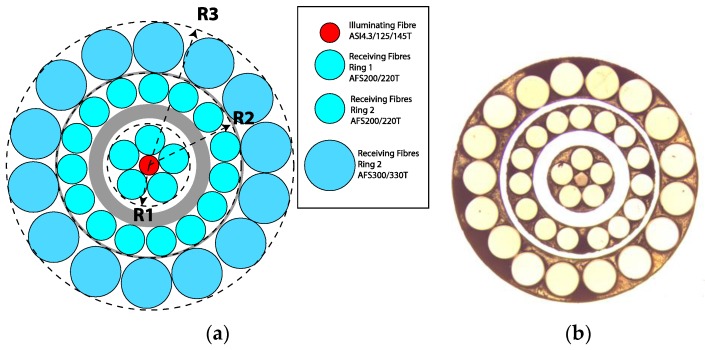
Initial design (**a**) of the bundle of optical fibers and a microscope picture (**b**) of the cross section of the common leg in the manufactured bundle.

Based on our previous experience, we finally decided to employ the fibers of the manufacturer Fiberguide Industries shown in Figure 5, where R1 = 300 µm, R2 = 700 µm, and R3 = 1070 µm. In the same figure, a microscope picture of the cross section of the manufactured bundle is also depicted. The final distances in our manufactured bundle were R1 = 324 µm, R2 = 686 µm, and R3 = 1125 µm, somewhat different from the design radius.

**Figure 6:**
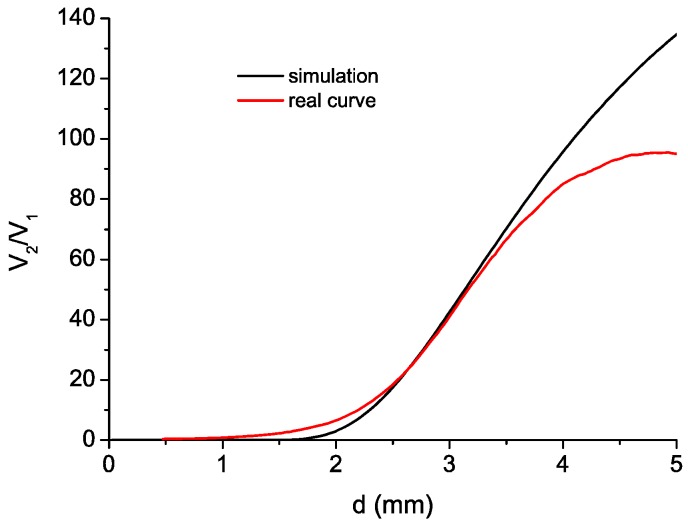
Simulated calibration curve and real calibration curve for the sensor using the tetrafurcated bundle.

Before manufacturing the bundle, we developed a MATLAB program in order to verify the behavior of the sensor. This software was developed according to the theoretical models described in literature [23,31,32,33]. As all these papers assume that the target is a mirror instead of a blade, we adjusted the parameters of the reflected light in order to get more realistic results. This parameter optimization was achieved by performing several experimental measurements using the trifurcated bundles we have in our laboratory. Figure 6 depicts the experimentally obtained calibration curve for the sensor employing the tetrafurcated bundle in the region I, together with the simulation. Table 1 shows the distance differences between the two curves for the same values of V_2_/V_1_ obtained along the measurement range. We can see that the simulation provides good results except for the last part of the measurement range, where the distance difference increases significantly.

**Figure 7:**
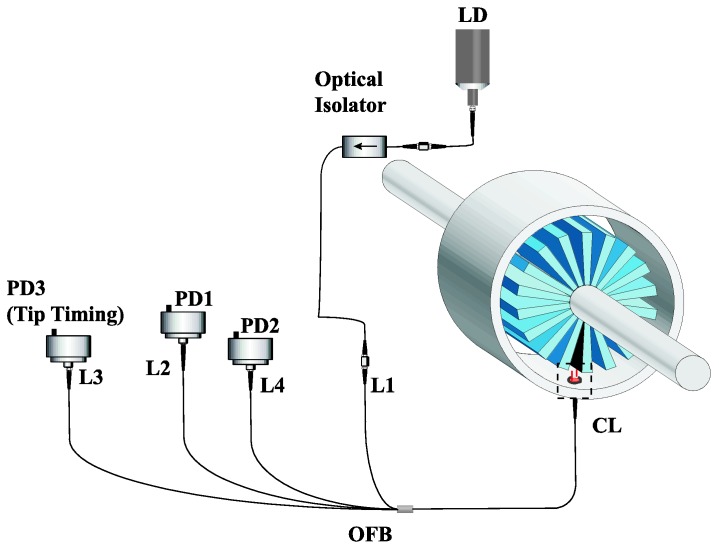
Components of the optical fiber sensor for TC measurements. LD stands for Laser Diode, PDx for Photodetector x, Lx for Leg x, CL for Common Leg and OFB for optical fiber bundle.

The rest of the components of the sensor are depicted in Figure 7. A laser module from Frankfurt Components (HSML-0660-20-FC, Frankfurt Laser Company, Friedrichsdorf, Germany) was employed as source of light. It has a nominal output power of 20 mW at 660 nm. An optical isolator (IO-F-660 from Thorlabs, Newton, NJ, US) was placed between the laser and the bundle in order to avoid any possible reflection that could destabilize the light source. Finally, the photodetectors employed were PDA100A-EC ones from Thorlabs. Photodetectors 1 and 2 were employed for TC measurements with transimpedance gains of 1.51 × 10^3^ V/A (BW = 1.5 MHz) and 1.51 × 10^5^ V/A (BW = 60 kHz), respectively. Photodetector 3 was used for tip timing measurements and its gain was 4.75 × 10^4^ V/A (BW = 200 kHz).

**Figure 8:**
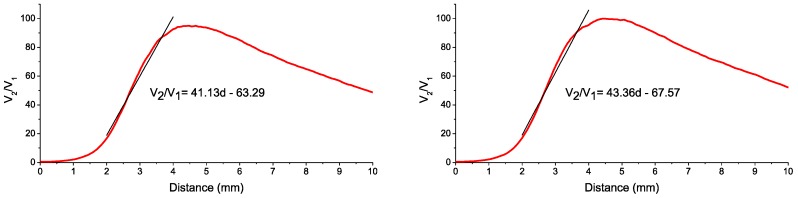
Calibration curves for 20 °C (**Left**) and 10 °C (**Right**).

Regarding the calibration of the sensor, we employed a linear translation stage and we followed a procedure similar to our previous works [20]. Since it was impossible to use a compressor blade for the calibration process, the most similar blade available in our laboratory was employed. The ambient temperature of the test cell was expected to be quite low (5–10 °C), so we checked the effect of the temperature on the calibration curve of the sensor. We introduced all the sensor components in a climate chamber and we carried out two calibrations at 20 °C and 10 °C. The resulting curves and their linearization for the measurement range are depicted in Figure 8. Even though the distance difference to the linearized calibration curve was quite small at the beginning of the measurement range, it reached 100 µm in the last part of the measurement range, so we employed the calibration obtained at 10 °C for the tests.

### 2.2. Experimental Set-up

**Figure 9:**
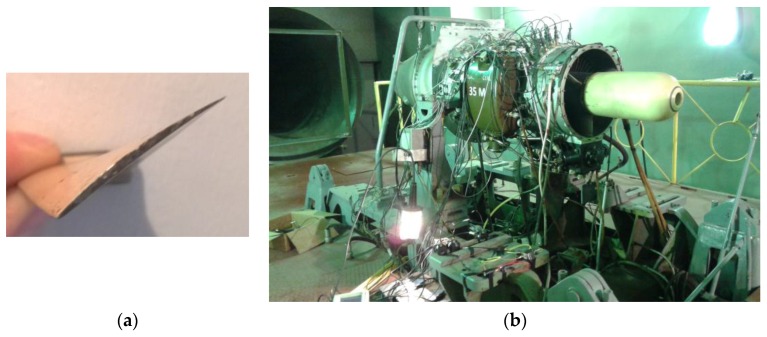
(**a**) Upper view of a blade of the first stage of the compressor; (**b**) SO-3 engine in the test cell with the optical sensor installed in its casing.

The performance of the optical sensor was tested in the SO-3 engine that is employed to power the TS-11 “Iskra” combat jet trainer. Its compressor is composed of seven stages and the optical sensor was installed in the first one. This stage has 28 blades made of 18H2N4WA steel, with a length of 100 mm, a chord of 37 mm, and a maximum width of 1.5 mm. The surface of the blade is rough and it usually presents some corrosion which makes the measuring more difficult. The tests were performed on the test cell that the Air Force Institute of Technology has in Warsaw. Figure 9 depicts the upper view of the blade and the engine in the test cell.

**Figure 10:**
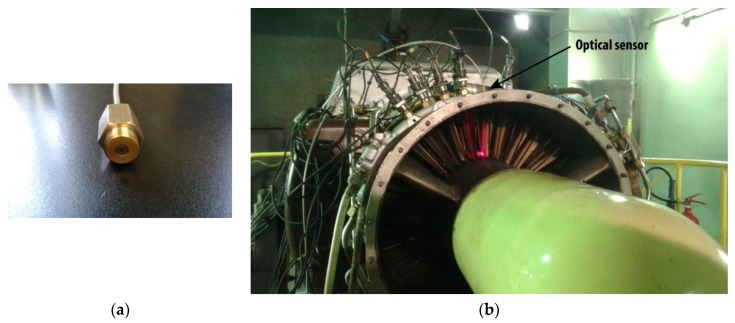
(**a**) Bracket employed to fix the bundle to the casing of the engine; (**b**) Bundle of optical fibers installed in the casing. Several inductive sensors for tip-timing measurements were also installed in the casing.

A special bracket was designed to fix the tip of the bundle in the casing of the engine. The tip of the bundle was placed at an approximated distance of 3 mm from the blade tip. Both the bracket and the final arrangement of the bundle in the area of the casing corresponding to the first stage of the compressor can be observed in Figure 10. At this part of the engine the gas temperature is approximately the same as the ambient temperature. The rotational speed of the engine is dependent on the operation condition of the engine, and the revolutions per minute (rpm) of the rotor range from 6900 rpm in idling condition to a maximum of 15,600 rpm during the takeoff.

**Figure 11:**
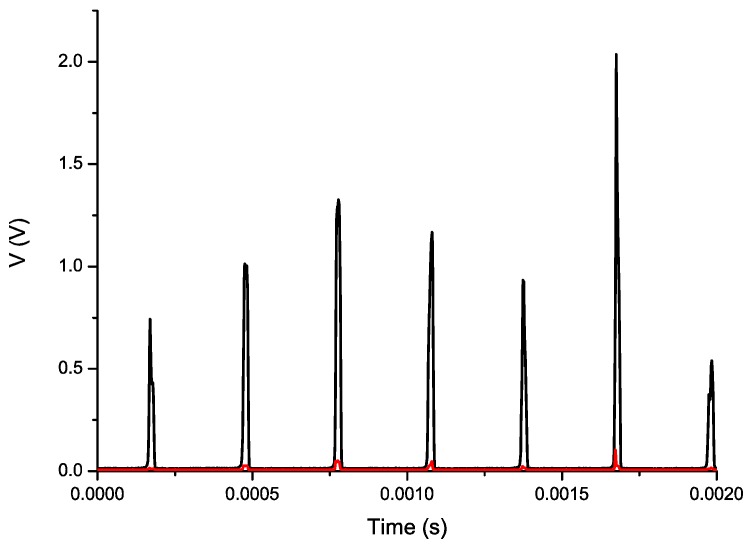
Signals obtained from both photodetectors, V_1_ (**Red**) and V_2_ (**Black**), when the engine was in idling state.

The signals provided by the photodetectors are very sharp due to the small thickness of the blades, and their amplitudes are quite different for each of the blades, as can be seen in Figure 11. Whereas V_1_ is around 100 mV, certain blades produce peaks of several volts in V_2_. Therefore, we had to use two different PXIe-6358 data-acquisition modules (from National Instruments, Austin, TX, United States) in order to take advantage of the 16-bit resolution of each module. The sampling frequency for the signal acquisition was 500 kS/s.

## 3. Results

**Figure 12:**
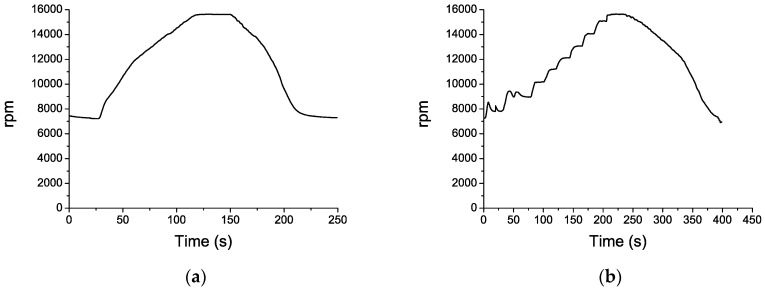
Flight profile for the cycles 1 (**a**) and 2 (**b**).

The tests consisted of four complete cycles in which the engine ran from idling operational state to takeoff and back to idling state again. The starting and final rotational speed was approximately 7000 rpm and the maximum speed reached during takeoff was 15,600 rpm. In our first and the third cycle the engine acceleration and deceleration were linear, the rotational speed increased continuously up to 15,600 rpm and then decreased back to idling state. In our second and fourth cycle the rotational speed increased in steps of about 1000 rpm (see Figure 12b), whereas the deceleration was linear as in the other cycles. The flight profile for the cycles 1 and 2 are shown in Figure 12.

**Figure 13:**
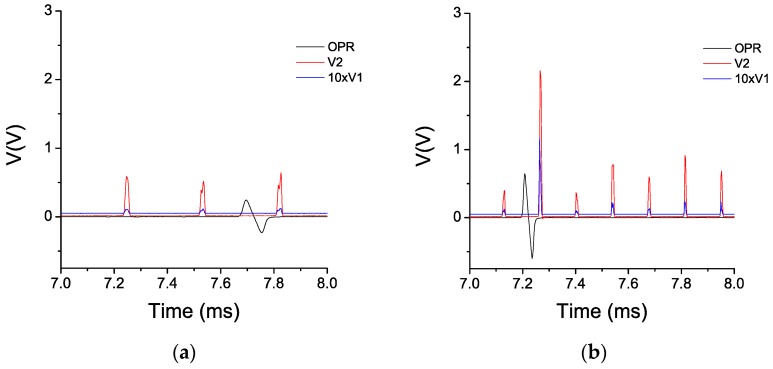
OPR, V_1_ (×10) and V_2_ signal during the Cycle 1 at 7600 rpm (**a**) and at 15,600 rpm (**b**).

During each cycle, three signals were acquired: the OPR (Once Per Revolution) signal and the signals corresponding to the outputs of the photodetectors, V_1_ and V_2_. These signals were stored and post-processed off-line using a LabVIEW program designed specifically for the analysis of TC measurements using the sensor. The program divides the whole acquisition in individual revolutions making use of the OPR signal. In this way, it can evaluate the quotient V_2_/V_1_ during one revolution and find the TC values for each of the 28 blades of the compressor. The minimum of such values is considered as the TC for that revolution. To calculate the TC value for each blade, the calibration curve in Figure 8 is employed to convert the value of the quotient V_2_/V_1_ into distance. In Figure 13 the three signals (OPR, V_1_ and V_2_) are depicted at 7600 rpm and at 15,600 rpm. For the sake of clarity, the amplitude of the signal V_1_ has been magnified by a factor of 10. In this figure, it is clearly seen that the amplitude of the signals becomes higher as the rotational speed increases. This is due to the fact that the faster the engine is turning, the closer the blades are to the casing and the higher is the reflected light intensity that reaches the bundle of optical fibers.

In Table 2 the TC measurements during the acceleration of the cycles 1 and 3 are shown. In order to calculate the TC at each rotational speed, an interval of 0.2 s (100,000 samples) was considered as a “constant-speed” interval. The rotational speed in each measurement is the average of the interval. In both cycles, the TC decreases as the rotational speed increases showing a reasonable behavior. The results for the TC values are quite similar during both acceleration cycles, being the most significant differences between 12,000 and 15,000 rpm. In the case of 15,000 rpm, the sensor shows a maximum difference of 96 µm.

**Figure 14:**
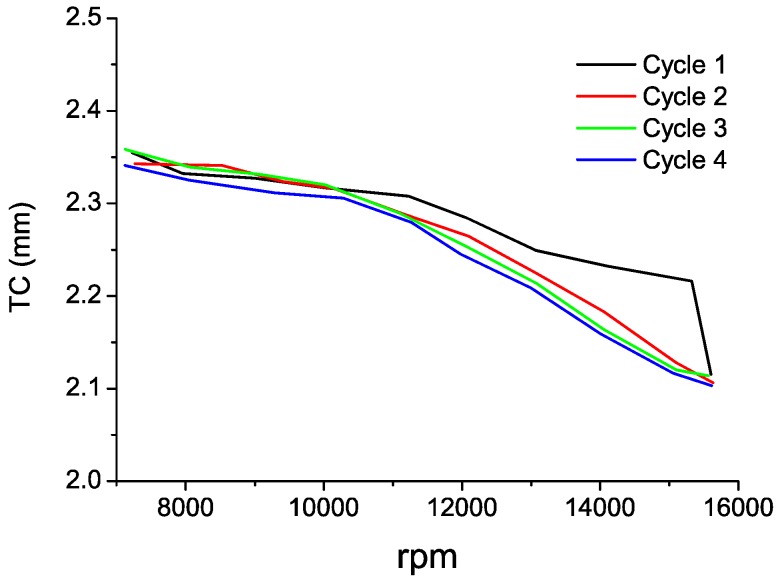
TC measurements in the four cycles during acceleration from idling state to takeoff.

In Table 3, we can observe the results during the acceleration of the cycles 2 and 4. In these cases the transition from idling state to takeoff is accomplished in steps of 1000 rpm approximately. These cases yield longer intervals in which the rotational speed is constant, so we decided to employ 0.6-s intervals (300,000 samples) to determine the TC at each speed. The behavior of the sensor is correct during both cycles, and the maximum TC difference between the cycles is 24 µm. In Figure 14, the measurements during the four cycles have been plotted. Except for the case of the first cycle in the range from 12,000 to 15,000 rpm, the TC measurement exhibits the same evolution in all cycles and the sensor provides similar TC values for all the speeds of each cycle.

**Figure 15:**
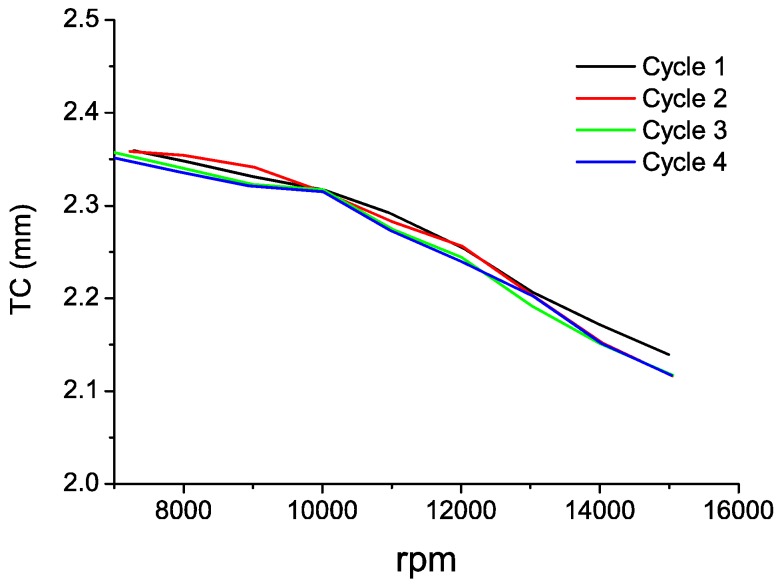
TC measurements in the four cycles during the deceleration from takeoff to idling state.

In Table 4, the TC values obtained during the engine deceleration in every cycle are depicted. For all cases, the deceleration is linear, and the evolution of the measurement is shown in Figure 15. As can be seen in the graph, the four cycles behave in a highly analogous way, yielding a maximum difference of 23 µm among the measurements, which is very similar to the previous case.

**Figure 16:**
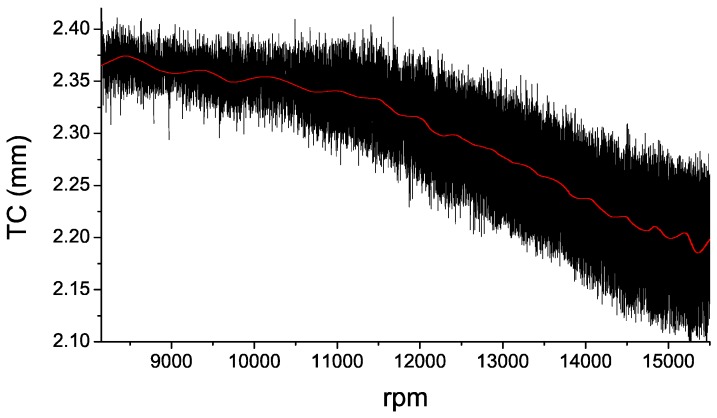
TC measurements for each revolution during the acceleration in the Cycle 3, and a smoothed representation of the results (**red line**).

**Figure 17:**
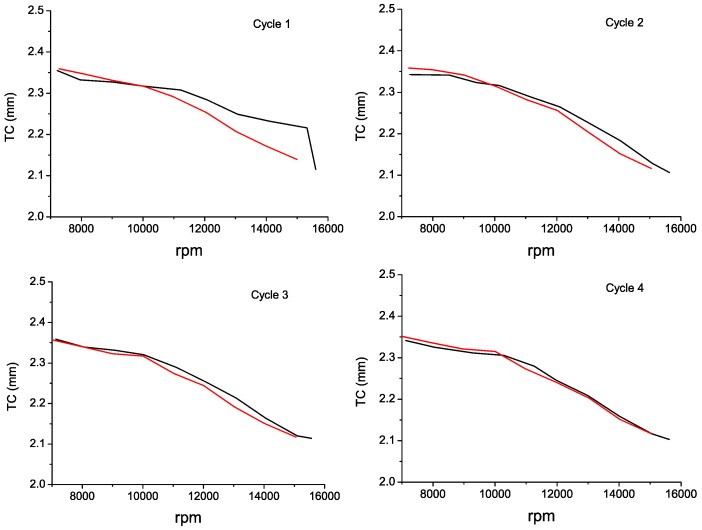
Acceleration (**Black**) and deceleration (**Red**) for each cycle of the engine operation.

In Figure 16, the TC values for each revolution of the acceleration in the Cycle 3 have been plotted. In order to help to visualize the TC evolution during the engine acceleration, a smoothed representation of the results is also shown. In Figure 17, the TC measurements of each cycle of acceleration and deceleration are depicted. These charts allow us to verify that there are no apparent signs of hysteresis in the performance of the sensor.

Regarding the accuracy of the results, unfortunately no other sensor could be installed to measure TC during the tests. The amplitude of the signals provided by the inductive sensors installed for tip timing measurement depended on speed, and they were not calibrated for tip clearance measurement. Consequently, there is not any reference with which the results can be compared. However, it is worth noting that the laboratory tests carried out prior to the measurements in the test cell with the real engine provided errors lower than 1% in the range of the measurement with a minimum resolution of 1 µm. With respect to the sensor precision, these preliminary tests give us a standard deviation in the laboratory measurements of 2 µm. In order to compare this value with the measurements in the real engine, the TC for each rotational speed has been assigned to nearest value of the rotational speed shown Table 5, in such a way that we have eight values for each rotational speed (except for 15,600 rpm, in which we have only four). As can be observed in Table 5, the maximum value for the standard deviation of all cycles is 34 µm. The Cycle 1 contributes significantly to heighten this value due to the strange behavior of the measurements during the acceleration of this cycle. If we discard this cycle, the maximum standard deviation drops to 12 µm, which is an excellent value if we take into account that the measurements were taken at slightly different rotational speeds.

## 4. Conclusions

The TC measurement for the first stage of a compressor of an aircraft engine was carried out using an optical fiber sensor. The engine in the test cell worked in real conditions. The tests consisted of four cycles from idling state to takeoff and back to idling state. Even though it was no possible to install a reference sensor to compare the accuracy of the results, the sensor showed a correct behavior as regards the variation of the rotational speed. The standard deviation of the measurements, if we discard the Cycle 1 due to its strange behavior in the last part of the acceleration, was 12 µm. This is a notable value if we take into account that the measurements were taken at slightly different speeds and that the conditions were adverse due to the corrosion present on the blades. It is also worth noting the short time needed to calibrate and to install the sensor in the engine, and the feasibility of performing independent tip-timing measurements using the leg three of the optical fiber bundle. In conclusion, a correct behavior of the sensor was demonstrated for the first stage of the compressor in such a way that we can expect similar results in harsher environments as the turbine. In order to get satisfactory results, the high temperature and contamination issues must be correctly tackled with the necessary modifications in the bundle design.

## Figures and Tables

**Table 1:** Comparison between distances obtained using the real and simulated calibration curve.

V_2_/V_1_	Simulation Distance (mm)	Experimental Distance (mm)	Difference (mm)
20	2.575	2.55	0.025 (1%)
30	2.775	2.78	−0.005 (−0.2%)
40	2.97	3	−0.03 (−1%)
50	3.15	3.175	−0.025 (−1.1)
60	3.325	3.375	−0.05 (−1.5%)
70	3.5	3.6	−0.1 (−2.8%)
80	3.7	3.875	−0.175 (−4.37%)
90	3.9	4.325	−0.425 (−10.9%)

**Table 2:** TC values during the acceleration of the cycles 1 and 3.

rpm	TC Cycle 1 (mm)	rpm	TC Cycle 3 (mm)
7226	2.355	7126	2.359
7960	2.332	8060	2.339
9003	2.327	9072	2.331
10,025	2.317	10,025	2.320
11,229	2.308	11,078	2.290
12,041	2.285	12,089	2.253
13,064	2.249	13,080	2.213
14,112	2.232	14,059	2.163
15,321	2.216	15,098	2.120
15,603	2.115	15,567	2.114

**Table 3:** TC values during the acceleration of the cycles 2 and 4.

rpm	TC Cycle 2 (mm)	rpm	TC Cycle 4 (mm)
7265	2.343	7124	2.341
8533	2.341	8051	2.325
9406	2.323	9293	2.311
10,147	2.316	10,284	2.306
11,174	2.288	11,259	2.280
12,092	2.265	11,980	2.245
13,068	2.225	12,997	2.209
14,055	2.183	14,006	2.159
15,096	2.128	15,047	2.117
15,635	2.106	15,617	2.103

**Table 4:** TC values during the deceleration.

**rpm**	**TC Cycle 1 (mm)**	**rpm**	**TC Cycle 2 (mm)**
14,998	2.139	15,049	2.116
14,001	2.171	14,039	2.152
13,030	2.207	13,028	2.203
12,050	2.253	12,009	2.256
10,941	2.293	10,996	2.283
10,028	2.317	10,006	2.315
9006	2.331	9024	2.342
8069	2.347	8004	2.354
7281	2.359	7226	2.358
**rpm**	**TC Cycle 3 (mm)**	**rpm**	**TC Cycle 4 (mm)**
15,055	2.117	15,039	2.117
14,007	2.151	14,028	2.151
13,022	2.192	13,043	2.202
11,997	2.245	11,997	2.240
11,014	2.274	10,980	2.273
10,006	2.317	10,006	2.315
9005	2.323	8942	2.321
8019	2.340	7962	2.336
6990	2.357	7011	2.351

**Table 5:** Mean and standard deviations for all the measurements.

rpm	Mean TC (mm)	Mean TC (mm) *	Standard Deviation (µm)	Standard Deviation (µm) *
7000	2.353	2.351	7	8
8000	2.339	2.339	9	9
9000	2.326	2.325	9	10
10,000	2.315	2.315	4	5
11,000	2.286	2.281	11	7
12,000	2.255	2.251	14	9
13,000	2.212	2.207	18	11
14,000	2.170	2.160	27	12
15,000	2.134	2.119	34	5
15,600	2.110	2.108	6	6

* Discarding Cycle 1.
